# Nintedanib combined with anticoagulant therapy in advanced lung cancer complicated with pulmonary fibrosis and pulmonary embolism: two case reports and clinical decision-making analysis

**DOI:** 10.3389/fcvm.2026.1771163

**Published:** 2026-04-10

**Authors:** Xiuli Zhou, Li Zhao, Wenming Ma, Baosheng Liu

**Affiliations:** 1 Department of Clinical Pharmacy, Weifang People's Hospital, Weifang, Shandong, China; 2 Pharmacy Department, China-Japan Friendship Hospital, Beijing, China; 3 Department of Clinical Pharmacy, Weifang Hospital of Traditional Chinese Medicine, Weifang, Shandong, China

**Keywords:** advanced lung cancer, anticoagulant therapy, individualized treatment, nintedanib, pulmonary embolism, pulmonary fibrosis

## Abstract

**Background:**

Lung cancer remains the leading cause of cancer-related deaths worldwide. With the development of targeted therapy and immunotherapy, the prognosis of patients with advanced non-small cell lung cancer has been significantly improved. However, the occurrence of comorbidities such as pulmonary fibrosis (PF) and pulmonary embolism (PE) significantly worsens the prognosis of these patients. Nintedanib is an effective antifibrotic drug, but its inhibition of vascular endothelial growth factor receptor may increase the risk of bleeding. Anticoagulant therapy is the cornerstone of PE treatment, and the combined use of nintedanib and anticoagulants may further increase the bleeding risk, which poses a clinical dilemma. At present, real-world data on the combined use of nintedanib and anticoagulants in patients with lung cancer complicated with PF and PE are scarce.

**Case presentation:**

We reported two cases of patients with advanced lung adenocarcinoma complicated with PF and PE. Case 1 was a 67-year-old male patient with left lung adenocarcinoma (cT2N2M1c IVB) complicated with idiopathic pulmonary fibrosis and acute PE. He had a history of hemoptysis after chemotherapy and was treated with nintedanib for antifibrosis before admission. After admission, nintedanib was temporarily discontinued due to the relative contraindication between anticoagulation therapy and bleeding. After the patient's condition stabilized, low-dose nintedanib was reinitiated, and the anticoagulant was switched to rivaroxaban orally after discharge. Follow-up showed no bleeding and the thrombosis disappeared. Case 2 was a 69-year-old male patient with right lung adenocarcinoma (cT4N3M1a IVA) with BRAF V600E and TP53 mutations, complicated with drug-related interstitial pneumonia and acute PE. He was treated with nintedanib combined with enoxaparin sodium. After the occurrence of mild hemoptysis, only the anticoagulant dose was adjusted without discontinuing nintedanib, and the bleeding symptoms were gradually relieved after discharge.

**Conclusion:**

The core contradiction in the treatment of advanced lung adenocarcinoma complicated with PF and PE is the balancing of bleeding risk between nintedanib antifibrotic treatment and anticoagulant therapy. Clinical decisions need to be based on individualized risk-benefit assessment. Individualized risk assessment and precise plan adjustment are key to improving the prognosis of these complex patients and providing practical references for similar clinical cases. Further studies are required in the future to confirm the safety of nintedanib when used in combination with anticoagulants.

## Introduction

Lung cancer remains the leading cause of cancer-related deaths worldwide, with a 5-year survival rate of only 10%–20% in the majority of countries. The development of targeted therapy and immunotherapy has led the treatment of lung cancer into the era of precision medicine, significantly improving the survival of patients with metastatic non-small cell lung cancer (NSCLC) (1, 2). However, the occurrence of comorbidities such as pulmonary fibrosis (PF) and pulmonary embolism (PE) significantly worsens the prognosis of these patients (3). PF is a chronic lesion involving interstitial lung tissue, leading to progressive damage of this tissue, a gradual decline in respiratory function, and a poor prognosis (4). Nintedanib inhibits key tyrosine kinase receptors closely related to fibrosis in the body and blocks the signal pathway for the abnormal proliferation and differentiation of fibroblasts and massive synthesis of extracellular matrices, such as collagen, induced by their overactivation, thereby reducing fibroblast activation and delaying the progression of pulmonary fibrosis; at the same time, it inhibits the release of inflammatory factors such as tumor necrosis factor-α (TNF-α) and interleukin-6 (IL-6), reduces the lung inflammatory response, and indirectly reduces the risk of inflammation-induced fibrosis (5, 6). However, the inhibition of vascular endothelial growth factor receptor (VEGFR) by nintedanib may be accompanied by an increased risk of bleeding. Clinical trials did not include patients with bleeding risks, such as congenital bleeding tendency or those receiving full-dose anticoagulant therapy. Therefore, the drug instruction suggests that it can be used in such patients only when the expected benefit outweighs the potential risk.

Moreover, the risk of venous thromboembolism (VTE)/PE in patients with advanced cancer is 4–7 times that of the general population and is closely related to a tumor-induced hypercoagulable state, limited patient activity, and treatment-related factors (7). Anticoagulant therapy, such as low-molecular-weight heparin (LMWH) or direct oral anticoagulants, is the cornerstone of PE treatment, but combined use with nintedanib may increase the risk of bleeding (8). The clinical dilemma is that discontinuing nintedanib may accelerate the progression of PF, while insufficient anticoagulation therapy increases the risk of PE recurrence. The Chinese Guidelines for the Diagnosis, Treatment, Prevention and Management of Pulmonary Thromboembolism (2025 Edition) emphasizes that individualized risk assessment and multidisciplinary collaboration are required for complex cases (9). However, current real-world data on the combined use of nintedanib and anticoagulants in patients with lung cancer complicated with PF and PE are relatively scarce. This study explores the logic of treatment decisions by reporting two typical cases combined with guidelines and clinical research evidence, providing a practical reference for clinical practice.

## Case presentation

### Case 1

A 67-year-old male patient (165 cm, 67 kg) was admitted to the hospital due to “left lung adenocarcinoma for more than 5 months, dyspnea for more than 4 months, and sudden shortness of breath with blood-tinged sputum.” Five months previously, he had been diagnosed with left lung adenocarcinoma (cT2N2M1c IVB) with multiple brain metastases by chest enhanced CT and endobronchial-ultrasound transbronchial needle aspiration (EBUS-TBNA) pathology. From 28 March to 15 July 2025, he regularly received chemotherapy, numbering five courses in total. The first three courses were bevacizumab 400 mg D1 + carboplatin 450 mg D2 + pemetrexed disodium 900 mg D2. Due to transient hemoptysis (approximately 50 mL) after three courses of chemotherapy, bevacizumab was discontinued in the subsequent two courses, and carboplatin + pemetrexed disodium chemotherapy was administered (specific dose unknown). He had a history of COVID-19 in 2023. Subsequent reexamination of chest CT indicated interstitial pneumonia, and he was diagnosed with “pulmonary fibrosis” for more than 5 months. He took nintedanib 100 mg bid orally for antipulmonary fibrosis treatment, and no bleeding occurred during the medication. He had a long-term smoking history and denied underlying diseases and allergies. Before admission, he suddenly developed dyspnea (minimum pulse oxygen 77%) and blood-tinged sputum (bright red, approximately 20 mouthfuls/day). An emergency ultrasound (18 July 2025) showed thrombosis in the bilateral femoral-popliteal veins, right posterior tibial vein, and left peroneal vein, with the widest part of the thrombus being 1.3 cm, and right calf muscular vein thrombosis. On 20 July, his D-dimer level was 13.37 mg/L. Suspected pulmonary embolism was considered. He was administered carbazochrome sodium for hemostasis and enoxaparin sodium for anticoagulant therapy. Considering the relative contraindication between hemoptysis and pulmonary embolism treatment, and that nintedanib may affect coagulation function, the drug was discontinued and the patient was transferred to the Department of Respiratory Medicine for further diagnosis and treatment. The admission diagnosis was as follows: Left lung adenocarcinoma (cT2N2M1c IVB), deep vein thrombosis of the lower extremities, suspected pulmonary embolism, type I respiratory failure, and idiopathic pulmonary fibrosis (Hamman–Rich syndrome).

After admission, the patient continued to receive enoxaparin (4,000 IU sc q12h) for anticoagulant therapy (21–23 July), and, at the same time, was administered carbazochrome sodium 60 mg intravenously qd (21 July to 1 August) combined with Yunnan Baiyao Capsules 0.5 g orally tid (21 July to 1 August) for hemostatic treatment. The patient had a cough and expectoration, and a pulmonary infection was considered. Empiric medication with ceftriaxone 2 g qd by intravenous drip was administered for anti-infection. Methylprednisolone sodium succinate 40 mg was administered by intravenous drip for antifibrotic therapy. Considering that the patient was currently using hormones, which posed a risk factor for *Pneumocystis jirovecii* pneumonia (PJP), 1 compound sulfamethoxazole tablet qd was administered to prevent PJP infection (10). His D-dimer level was checked on 21 July, which was 9.08 mg/L. On 23 July, pulmonary CTA showed pulmonary embolisms in the lingular segment of the left upper lobe, the right middle lobe, and the basal segment of the right lower lobe. A ventilation-perfusion (V/Q) scan revealed impaired blood perfusion but normal ventilation in these segments, which was considered post-treatment changes due to pulmonary embolism. On admission, the B-type natriuretic peptide (BNP) marker was higher than the normal values, while echocardiography showed no obvious abnormalities. The patient was graded as moderate-low risk for pulmonary embolism, and the dose of enoxaparin was adjusted to 6,000 U in the morning + 8,000 IU in the evening by subcutaneous injection.

On 24 July, a lower extremity venous color Doppler ultrasound still indicated a thrombus (maximum width of 1.1 cm), with no significant change compared with the previous results. Meanwhile, the patient’s D-dimer level was found to be 4.82 mg/L. Therefore, a consultation with a clinical pharmacist was requested on 25 July. After conducting a bedside inquiry, the pharmacist noted that the patient had no further hemoptysis. The patient had been on regular anticoagulant therapy for over a week, but the lower extremity ultrasound indicated that the anticoagulant effect was not significant. Following an increase in the dose of low-molecular-weight heparin, the patient’s D-dimer level decreased significantly, while his activated partial thromboplastin time (APTT) increased significantly to 48.5 s. Furthermore, the patient’s hemoglobin, platelet count, and liver and kidney functions were all normal. The patient's American College of Chest Physicians (ACCP) bleeding risk score was high (with four risk factors), and nintedanib could further increase the bleeding risk. However, considering the patient's pulmonary embolism combined with a high bleeding risk, it was recommended that after the low-molecular-weight heparin reached a stable state, if no re-hemoptysis occurred, the combined use of nintedanib 100 mg bid could be considered. Furthermore, after combined medication, the dose of enoxaparin could be appropriately reduced to 6,000 IU q12h, with close monitoring of bleeding tendency during this period. The attending physician adopted the clinical pharmacist's suggestion. The patient received enoxaparin (8,000 IU + 6,000 IU) until 1 August, with no complaint of bleeding manifestations; his D-dimer level decreased further and his general condition was stable, so the combined use of nintedanib 100 mg bid was started. However, considering that the patient was still in the acute phase of thrombosis, he was advised to continue subcutaneous injections of enoxaparin 6,000 IU q12h after discharge until 13 August 2025, and then directly switch to oral rivaroxaban 20 mg qd for anticoagulation therapy. During this period, close observation of systemic bleeding tendency was required; if symptoms such as gingival bleeding, melena, or aggravated hemoptysis occurred, timely medical treatment should be sought. Follow-up 1 month later showed no bleeding events, the thrombus had disappeared on re-examination, and dyspnea was improved.

### Case 2

A 69-year-old male patient (168 cm, 67 kg) was admitted for a 5-month history of right lung adenocarcinoma and a 3-month cough with dyspnea. Over 5 months prior, he had been diagnosed with right lung adenocarcinoma (cT4N3M1a IVA), with molecular pathology revealing BRAF p.V600E and TP53 p.R335Afs*8 mutations. He received oral targeted antitumor therapy with dabrafenib (150 mg bid) plus trametinib (2 mg qd) from 4 March to 7 April 2025. Three months before admission, he developed chest distress, shortness of breath, and dyspnea without obvious causes, accompanied by a cough and scanty expectoration. A chest CT on 14 April 2025 showed markedly reduced density of a mass-like hyperdense shadow in the right lower lung, decreased bilateral pulmonary lucency, and newly appeared ground-glass opacities with linear and fine reticular hyperdensities in both lungs, along with mild local bronchial traction bronchiectasis (predominant in bilateral lower lungs). Imaging confirmed newly developed bilateral interstitial pneumonia complicated with interstitial pulmonary fibrosis, as well as multiple enlarged lymph nodes in the mediastinum and right lung hilum. For the pulmonary fibrosis, he was administered methylprednisolone sodium succinate (40 mg ivggt q12h), which was subsequently switched to oral prednisone acetate tablets (20 mg q12h) with a gradual tapering regimen (the patient had no clear recollection of the specific tapering regimen). On 8 June 2025, he was readmitted to the local hospital due to a recurrent cough and dyspnea, and received further antifibrotic treatment with methylprednisolone sodium succinate (the specific dosage was not available). This therapy was subsequently switched to oral prednisone acetate at 35 mg once daily, which was gradually tapered to a maintenance dose of 20 mg once daily. Bronchoalveolar lavage fluid (BALF) next-generation sequencing (NGS) on 9 June 2025 identified *P. jirovecii* (relevant details unobtainable from the patient's past medical records and the family) and his Galactomannan (GM) test result was >0.87. Thus, the local hospital suspected PJP and administered trimethoprim-sulfamethoxazole (five tablets three times daily) for treatment. A follow-up chest CT half a month later showed clinical improvement. On 19 July, the patient revisited the local hospital, where pulmonary fibrosis due to oral targeted antitumor agents was suspected. He was therefore advised to discontinue the targeted antitumor therapy, and oral nintedanib esilate soft capsules (150 mg twice daily) were added for antifibrotic treatment. Hyperglycemia developed during steroid therapy, managed with subcutaneous insulin aspart (4 U before meals) and insulin glargine (8 U at bedtime). Prior to admission, the patient had no obvious improvement in chest distress and shortness of breath. Furthermore, he had family-reported poor fingertip oxygen saturation (96% at rest with 3 L/min nasal oxygen, dropping to 76%–88% on activity), leading to admission for further management. He remained conscious with a poor mental status; his diet and sleep were fair, and there was no significant weight loss. Physical examination showed a barrel chest, diminished bilateral breath sounds, and a small amount of moist and dry rales. He had a 40-year smoking and alcohol history (he had quit both) and construction dust exposure. Thus, the admission diagnoses were as follows: right lung adenocarcinoma (cT4N3M1a ⅣA), interstitial pneumonia, and hyperglycemia.

After admission, the patient continued to receive oral prednisone tablets (20 mg qd), esomeprazole enteric-coated tablets (20 mg qd), and nintedanib esilate soft capsules (150 mg q12h). Given his history of PJP and current glucocorticoid use, trimethoprim-sulfamethoxazole tablets were prescribed for PJP prophylaxis (10). His D-dimer level at this time was 18.56 mg/L. Lower extremity venous ultrasonography indicated calf muscular vein thromboses in both lower extremities. Chest contrast-enhanced CT (CECT) showed multiple filling defects in the pulmonary arteries, suggesting acute pulmonary embolisms (APEs). The patient was hemodynamically stable with an elevated serum NT-proBNP level, while echocardiography showed no obvious abnormalities. The patient was graded as intermediate-low risk for APE. Anticoagulant therapy was initiated for the APEs, and bronchoscopy was not performed at this time. Symptomatic treatments, including bed rest, anticoagulation therapy, and oxygen therapy, were administered, and oral targeted antitumor therapy was planned to be resumed after the stabilization of the PE. A subcutaneous injection of enoxaparin (6,000 IU sc q12h) for anticoagulation therapy was performed on 22 July.

On 24 July, a consultation with a clinical pharmacist was requested to assess whether the patient should continue nintedanib treatment. Based on the patient's various examination and test results, as well as his current condition, the clinical pharmacist considered that factors such as the patient's malignant tumor and long-term hormone therapy would further increase the risk of hypercoagulability. Although the patient's ACCP score for bleeding risk was high (with three risk factors), the patient had no previous bleeding history, normal liver and kidney function, and normal hemoglobin and platelet counts. Therefore, the bleeding risk was relatively controllable compared with the pulmonary embolism, and it was recommended to maintain the current treatment. Close monitoring of the patient's routine blood tests and coagulation function was required during the concomitant use, and drug adjustment would need to be made if bleeding occurred. The attending physician adopted the clinical pharmacist's suggestion.

On 28 July, the patient reported bright red hemoptysis (5–10 sputa streaked with blood per day) accompanied by exertional chest tightness and dyspnea, without other discomfort. Physical examination findings were as follows: nasal cannula oxygen inhalation at 3 L/min, peripheral oxygen saturation fluctuating at 98% and decreasing to 88% after activity; coarse breath sounds with a small amount of moist and dry rales in both lungs; no edema in either lower extremity. A recheck of coagulation function showed a fibrinogen level of 4.4 g/L, APTT of 48.2 s, and D-dimer level of 2.67 mg/L (other indicators were unremarkable). The decreased D-dimer level suggested the current anticoagulant therapy was effective and the patient's bleeding risk was controllable. Thus, the anticoagulant regimen was adjusted to subcutaneous enoxaparin sodium 6,000 IU in the morning and 4,000 IU in the evening, and Yunnan Baiyao was administered for hemostasis.

On 30 July, the patient still had a paroxysmal cough and expectoration with improvement compared with before, dark red hemoptysis with reduced volume, and persistent exertional chest tightness and dyspnea; his other conditions remained unchanged. Considering the patient's stable general condition, the patient was discharged and prescribed oral rivaroxaban 15 mg q12h for outpatient treatment (adjusted to 20 mg qd starting from 11 August). The patient was to continue taking nintedanib esilate soft capsules for antipulmonary fibrosis therapy. Close monitoring for bleeding was required during the treatment course, and immediate medical attention should be sought if bleeding symptoms exacerbate. After the patient's condition stabilizes, we will evaluate whether to continue oral targeted drug therapy (11).

## Discussion

PF is a group of chronic interstitial lung diseases (ILDs) characterized by excessive deposition of collagen in the lung interstitium, destruction of alveolar structures, and progressive decline of lung function. It is irreversible and highly fatal, with an average survival period of only 3–5 years (4). Its pathogenic factors are complex, including environmental exposure, drug toxicity, and viral infection. Among this group, drug-induced pulmonary fibrosis (DIPF) is induced by drugs such as bleomycin, amiodarone, and methotrexate. Post-COVID-19 pulmonary fibrosis is a common sequela in patients with severe COVID-19. Both have become clinical problems due to limited targeted treatment methods (12, 13). At present, only pirfenidone and nintedanib are approved for the treatment of idiopathic pulmonary fibrosis (IPF). The former works through anti-inflammation, antioxidation, and regulation of growth factors, while the latter, as a multitarget receptor tyrosine kinase (RTKI) inhibitor, can simultaneously inhibit PDGFR, FGFR, VEGFR, etc., and directly block the core signal pathway of fibroblast activation (14). Compared with pirfenidone, nintedanib shows consistent efficacy in non-IPF and IPF, which provides a theoretical basis for its expansion to DIPF and post-COVID-19 pulmonary fibrosis. Different from traditional anti-inflammatory drugs, the efficacy of nintedanib does not depend on inhibiting the early inflammatory response, but directly targets the progressive stage of fibrosis. Therefore, it delays the deterioration of fibrosis after drug withdrawal, providing a new strategy for postinjury intervention of DIPF (15).

Both patients had indications for using nintedanib to combat pulmonary fibrosis, but both developed acute pulmonary embolisms (moderate to low risk) requiring the initiation of anticoagulant therapy. Considering that concurrent use of nintedanib may cause bleeding, clinical pharmacists were requested to participate in evaluating whether to combine nintedanib during anticoagulation therapy. The clinical characteristics of the two patients are shown in Table 1.

**Table 1:** Clinical profiles, antithrombotic management, and outcomes of the two cases.

Item	Case 1	Case 2
Baseline characteristics	Male, 67 years old, 165 cm/67 kg; left lung adenocarcinoma (cT2N2M1c, stage IVB) with multiple intracranial metastases; pulmonary fibrosis for 5 months; long-term smoking history; no underlying diseases or drug allergies	Male, 69 years old, 168 cm/67 kg; right lung adenocarcinoma (cT4N3M1a, stage IVA) with BRAF p.V600E and TP53 p.R335Afs*8 mutations; pulmonary fibrosis for 3 months; previous *Pneumocystis jirovecii* pneumonia (PJP) infection; 40+ years of smoking and alcohol history (both ceased); chronic exposure to building dust; history of glucocorticoid therapy; Glucocorticoid-induced Hyperglycemia
Reason for admission	Shortness of breath accompanied by blood in sputum	Chest tightness, shortness of breath, and a significant decrease in finger pulse oxygen after activity
Pulmonary embolism risk stratification	Moderate-to-low risk	Moderate-to-low risk
Key risk factors	1. Age ≥65 years; 2. Malignant tumor with distant metastases; 3. Previous hemoptysis induced by bevacizumab; 4. History of nintedanib use	1. Age ≥65 years; 2. Malignant tumor with metastases; 3. Long-term glucocorticoid therapy
Initial interventions	Discontinuation of nintedanib; initial enoxaparin sodium 4,000 IU q12h, adjusted to 6,000 IU in the morning + 8,000 IU in the evening	Oral prednisone 20 mg qd, nintedanib 150 mg bid; enoxaparin sodium 6,000 IU q12h
ACCP bleeding risk score	High risk (age >65 years; previous bleeding history; malignant tumor; metastatic tumor)	High risk (age >65 years; malignant tumor; metastatic tumor)
Purpose of consultation	To evaluate whether to restart nintedanib	To evaluate whether to discontinue nintedanib
Consultation opinion	Restart nintedanib (100 mg bid) and reduce the dose of low-molecular-weight heparin to 6,000 IU q12h after hemoptysis stabilizes	Maintain current treatment
Clinical outcome	No hemoptysis occurred; sequentially switched to oral rivaroxaban 20 mg qd; follow-up thrombosis workup was favorable	Hemoptysis with blood in sputum occurred; improved after reducing the dose of low-molecular-weight heparin (6,000 IU in the morning + 4,000 IU in the evening); sequentially switched to oral rivaroxaban 15 mg bid

VEGFR-TKIs, including nintedanib, have been confirmed in multiple studies to be associated with an increased risk of bleeding. A network meta-analysis encompassing 50 randomized controlled trials with a total of 16,753 patients with cancer showed that compared with the control group (standard chemotherapy or placebo), VEGFR-TKIs including nintedanib were associated with a significantly higher incidence of all-grade bleeding events (OR = 1.79, 95% CI: 1.50–2.13, *P* < 0.0001), whereas no significant difference was observed in the risk of high-grade (Grades 3–5) bleeding (OR = 1.22, 95% CI: 0.87–1.71, *P* = 0.74) (16). The increased bleeding risk is a direct consequence of VEGF/VEGFR pathway inhibition. VEGF is generally understood to exert a stimulatory effect on the proliferation of vascular endothelial cells, which promotes endothelial cell survival and maintains vascular integrity, thereby ensuring the normal regulatory function of the coagulation system. In contrast, inhibition of the VEGF/VEGFR signaling pathway reduces the regenerative capacity of vascular endothelial cells, leading to the exposure of procoagulant phospholipids beneath the vascular matrix and impairment of platelet function, which in turn results in bleeding or thrombosis (17, 18). A reanalysis of a meta-analysis focusing on VEGFR-TKIs demonstrated that nintedanib was associated with an elevated bleeding risk, yet no significant correlation was found with serious cardiovascular events such as thrombosis, myocardial infarction, and stroke (19).

When we compared data from six clinical trials of nintedanib for idiopathic pulmonary fibrosis (20) with the pooled 9-year global postmarketing data (21), we found that diarrhea was the most common adverse event of nintedanib, with a low incidence of cardiovascular events, and its overall safety profile was manageable. For bleeding events, the incidence in the pooled clinical trial group (9.3 per 100 patient-exposure years) was lower than that in the INPULSIS (22) trial group (15.8 per 100 patient-exposure years) and there was no significant difference from the placebo group (10.2 per 100 patient-exposure years). This indicates that the bleeding risk became more stable with an expanded sample size (1,126 vs. 638 patients) and prolonged follow-up (a maximum of 93.1 months vs. 52 weeks). Mild to moderate local bleeding (e.g., epistaxis, hemoptysis) predominated across all trial groups. Postmarketing data showed that the incidence of bleeding events (2.42 per 100 patient-exposure years) was lower than that in all clinical trial groups. Even though the postmarketing cohort included users of anticoagulant/antiplatelet agents who were excluded from clinical trials, no increased bleeding risk was observed. However, new types of mild bleeding, such as ecchymosis and hematochezia, were reported, among which 81.3% were non-serious events and fatal events accounted for only 1.4% (mainly gastrointestinal bleeding and intracerebral hemorrhage). Moreover, 53% of bleeding events occurred within the first 3 months of treatment, which may be associated with vascular endothelial adaptation during the initial phase of drug administration. In addition, 64.9% of the patients were aged ≥65 years, including a large proportion of individuals with comorbidities (e.g., hypertension, coronary heart disease), and 19.8% of the patients with bleeding events were receiving concomitant anticoagulant therapy. For thrombus-related events (myocardial infarction, ischemic stroke, and venous thromboembolism), the clinical trials did not individually report the incidence of ischemic stroke and venous thromboembolism, with only data on myocardial infarction incidence included. This suggests that ischemic stroke and venous thromboembolism had an extremely low incidence in the clinical trials and thus were not a primary focus. Postmarketing data revealed that the incidence of myocardial infarction was lower than that in the clinical trial groups, and the majority of the affected patients had underlying cardiovascular diseases such as hypertension, coronary heart disease, and diabetes mellitus, indicating that such events were more closely associated with patients' comorbidities. Furthermore, the incidences of ischemic stroke and venous thromboembolism were 0.33 and 0.2 per 100 patient-exposure years, respectively, both lower than the incidence of bleeding events (2.42 per 100 patient-exposure years). An analysis of the differential incidence of bleeding and thrombus events with nintedanib suggests that this may stem from the following factors. First, there was selection bias in the study population: the clinical trials strictly excluded patients at high risk of bleeding, users of anticoagulant/antiplatelet agents, and those with a recent history of thrombosis, resulting in a highly homogeneous study cohort. In contrast, postmarketing use covers a broader range of indications and includes high-risk populations with hypertension, coronary heart disease, and other comorbidities, making the sample more reflective of real-world clinical practice. Second, the increased sample size and longer follow-up duration diluted the incidence of such events. Third, the clinical trials implemented rigorous standardized monitoring with documentation of all mild events, whereas postmarketing data are subject to underreporting of non-serious events; however, clinicians promptly adjust doses or discontinue the drug in clinical practice, which reduces the incidence of serious events. Fourth, there are different clinical dosing scenarios: the clinical trials mainly used a fixed dose of 150 mg bid, while postmarketing use includes a 100 mg bid dose group with more flexible dose adjustments.

In addition, rare case reports have indicated a potential association between nintedanib and intramural aortic hematoma. Although no population-based data support this link, it prompts the need to screen for such risks in patients presenting with acute chest and back pain (23). Multiple reports have shown that nintedanib may induce acute severe thrombocytopenia with or without hypofibrinogenemia, yet all these abnormalities are reversible following drug discontinuation (24–26). Overall, the bleeding risk of nintedanib is manageable. In clinical practice, monitoring for common bleeding types is required; enhanced follow-up should be conducted for patients at high bleeding risk, and monitoring for rare severe bleeding events is necessary to ensure that the benefits outweigh the risks.

For patients with confirmed acute low-to-moderate-risk pulmonary embolisms, anticoagulation therapy should be initiated as early as possible (9). However, how does one make clinical decisions when combining nintedanib with anticoagulants? No patients receiving anticoagulants were included in previous clinical trials, resulting in a temporary lack of data on the bleeding risk of nintedanib in combination with anticoagulant therapy. A real-world study involving 2,794 patients showed that the overall incidence of bleeding in patients with IPF was only 0.29%, with 0.25% in the nintedanib group—significantly lower than clinical expectations—and no bleeding events were even observed in the subgroup receiving nintedanib combined with anticoagulants alone. Even in the subgroup with concomitant anticoagulant and antiplatelet therapy, the bleeding incidence (0.81 per 100 patient-exposure years), though higher than that in other subgroups, showed no statistically significant difference (*P* = 0.072). Among the eight bleeding events, seven were mild to moderate and only one was severe gastrointestinal bleeding. These findings demonstrate that the bleeding risk of nintedanib combined with anticoagulants or antiplatelet agents is generally manageable. Nevertheless, it reminds us that enhanced monitoring is warranted for patients aged ≥75 years, those with ≥2 cardiovascular comorbidities (e.g., hypertension, coronary heart disease and atrial fibrillation), and patients with abnormal liver and renal function (8). Another retrospective study reported no bleeding events in patients with IPF receiving oral anticoagulants concomitantly with nintedanib, which further supports the safety of this combination in real-world clinical settings (27). The bioavailability of nintedanib at the standard therapeutic dose (150 mg bid) is only approximately 5%, exerting mild interference with the coagulation system in practice. Studies have shown that direct oral anticoagulants (DOACs) have a more favorable safety and tolerability profile than vitamin K antagonists, and thus can be regarded as the preferred regimen for concomitant anticoagulation therapy (28). Discontinuation of nintedanib is not routinely required when bleeding occurs during treatment; instead, an individualized judgment should be made based on the severity and type of bleeding and the patient's overall condition. For mild bleeding, symptomatic management is the mainstay without drug discontinuation. For clinically relevant or severe bleeding, a temporary drug hold is needed, and the decision to restart nintedanib should be made after comprehensive evaluation; permanent discontinuation is indicated for fatal bleeding (29).

Accurate assessment of bleeding risk is crucial for the standardized treatment of VTE. Selecting an appropriate bleeding risk assessment model or scale can help clinicians develop safer and more effective anticoagulation regimens. Current evidence suggests that anticoagulation therapy is relatively safe in populations stratified as low bleeding risk by the ACCP or VTE-BLEED (9). The ACCP bleeding risk stratification schema (30), proposed by the ACCP in 2012, is primarily used to assess the bleeding risk in patients with VTE, especially the risk of major bleeding during anticoagulation therapy. This score includes 18 bleeding risk factors and stratifies patients into low, moderate, or high bleeding risk categories following anticoagulation therapy. Since this model covers risk factors comprehensively and has appropriate accuracy in validation studies, it is mainly used for inpatients. The VTE-BLEED (31) was developed by Klok et al. in 2016 based on a cohort of patients with VTE who underwent anticoagulation therapy with dabigatran and warfarin. It is mainly used to predict the risk of major bleeding in patients with VTE who have completed acute-phase treatment and entered the long-term anticoagulation phase, and has limited predictive value for acute-phase anticoagulation therapy. In addition, new models such as the PE-SARD (32) have shown promising application prospects. Overall, however, the core limitations of all bleeding assessment scales include limited predictive accuracy and significant design variations. In clinical application, an appropriate scale should be selected, dynamic and repeated assessments should be performed, and interventions should focus on controllable risks. Clinical judgment and patient preferences should also be integrated. Notably, high bleeding risk is not a reason for drug discontinuation, but a signal for treatment optimization (33).

Based on the present study, the ACCP score was more appropriate for the two included patients. After evaluation, both patients were classified as high risk of bleeding within 0–3 months. Therefore, controllable factors that may cause bleeding, such as drug selection and adjustment of anticoagulant dosage, should be carefully monitored during anticoagulation therapy. Although both cases shared three risk factors (advanced age, malignant tumor, and tumor metastasis), the bleeding risk in Case 1 was further elevated due to a history of previous bleeding. This was mainly associated with hemoptysis induced by previous bevacizumab administration. The package insert for bevacizumab emphasizes that bevacizumab should not be used in patients with recent pulmonary hemorrhage/hemoptysis (>1/2 teaspoon of bright red blood), so the discontinuation of bevacizumab in this patient was appropriate. However, the theoretical half-life of bevacizumab is up to 20 days in male patients, and the risk of bleeding remains for 3–5 half-lives even after drug discontinuation. This patient was admitted with a pulmonary embolism complicated by hemoptysis, so bevacizumab may have been a potential contributing factor to the hemoptysis. At that time, the attending physician discontinued nintedanib out of concern that it may further exacerbate bleeding, and focused on the treatment of the acute pulmonary embolism and symptomatic management of hemoptysis. Subsequently, the patient had no recurrent hemoptysis, and his hemoglobin and platelet counts remained stable within the normal range; the pulmonary embolism was effectively managed with adjusted LMWH dosage. On this basis, clinical pharmacists considered that the bleeding risk of restarting nintedanib was manageable for this patient (27–29) and thus recommended the combination of nintedanib with anticoagulation therapy. In addition, it is noteworthy that the occurrence of VTE in this patient was consistent with the temporal relationship of bevacizumab administration and is also one of the common adverse events of bevacizumab. Therefore, monitoring for thrombosis is also required in patients receiving bevacizumab; timely assessment of thrombotic and bleeding risks and appropriate management measures should be adopted (34). A multicenter retrospective study showed that in patients with cancer with cancer-associated thrombosis (CAT) during bevacizumab treatment, continued bevacizumab administration during anticoagulation therapy was not associated with an increased incidence of bleeding complications or recurrent thromboembolism, though this conclusion requires validation in larger sample size studies (35). For Case 2, both bleeding and thrombotic risks were primarily driven by non-modifiable factors such as age and malignancy. However, long-term glucocorticoid use may have exacerbated the patient's hypercoagulable state, which was unfavorable for a pulmonary embolism. Although subsequent combination therapy with standard-dose nintedanib led to hemoptysis, his hemoglobin and platelet counts remained stable, while his D-dimer levels decreased significantly, indicating effective anticoagulation. Hemoptysis improved after dose adjustment of low-molecular-weight heparin, which is consistent with guideline recommendations (9).

A comparison of the two patients revealed the following: Case 1 was admitted with a pulmonary embolism complicated by hemoptysis and had a previous history of hemoptysis; after the resolution of hemoptysis, no bleeding events occurred with nintedanib combined with LMWH anticoagulation therapy. In contrast, Case 2 had no bleeding at admission and no previous bleeding history, but developed hemoptysis after nintedanib was combined with LMWH. In Case 1, who had multiple bleeding risk factors, dose reduction of nintedanib may have reduced the incidence of bleeding to a certain extent, while Case 2 received standard-dose nintedanib combined with the same dosage of LMWH. This suggests that different dosages of nintedanib may have varying effects on bleeding risk, though this finding requires further validation in additional studies.

## Conclusion

The core therapeutic dilemma in the management of advanced lung adenocarcinoma complicated by PF and PE lies in balancing the bleeding risk associated with nintedanib-based antifibrotic therapy and anticoagulation therapy, and clinical decision-making should be grounded in an individualized risk-benefit assessment.

This study is a single-center case report of two patients, with inherent limitations including a small sample size, absence of statistical power, and lack of a control group, which renders the study’s findings non-generalizable. Furthermore, as they were constrained by limited follow-up duration and individualized clinical management, the findings only reflect short-term clinical observations, and the relevant conclusions still warrant further validation through multicenter studies with a large sample size.

## Data Availability

The original contributions presented in the study are included in the article/Supplementary Material, further inquiries can be directed to the corresponding author.
